# Prevalence and risk factors for Chlamydia trachomatis seropositivity and seropersistence among women: A prospective cohort study

**DOI:** 10.1371/journal.pone.0328449

**Published:** 2025-08-05

**Authors:** Zoïe W. Alexiou, Bernice M. Hoenderboom, Christian J. P. A. Hoebe, Iris Scholte, Reshtrie Sheombarsing, Nicole H.T.M. Dukers-Muijrers, Birgit H.B. van Benthem, Servaas A. Morré

**Affiliations:** 1 Centre for Infectious Disease Control, National Institute for Public Health and the Environment, Bilthoven, The Netherlands; 2 Institute for Public Health Genomics (IPHG), GROW Research Institute for Oncology and Reproduction, Maastricht University, Maastricht, the Netherlands; 3 Department of Social Medicine, Care and Public Health Research Institute (CAPHRI), Maastricht University, Maastricht, The Netherlands; 4 Department of Sexual Health, Infectious Diseases and Environmental Health, Living Lab Public Health Mosa, Public Health Service South Limburg, Heerlen, The Netherlands; 5 Department of Medical Microbiology, Infectious Diseases and Infection Prevention, National Chlamydia trachomatis Reference Laboratory, Care and Public Health Research Institute (CAPHRI), Maastricht University Medical Center (MUMC+), Maastricht, the Netherlands; 6 Department of Health Promotion, Care and Public Health Research Institute (CAPHRI), Maastricht University, Maastricht, the Netherlands; UTHSCSA: The University of Texas Health Science Center at San Antonio, UNITED STATES OF AMERICA

## Abstract

**Introduction:**

Population-based *Chlamydia trachomatis* (CT) serology studies help evaluate the effectiveness of CT-control strategies. Determinants of CT seropersistence over time are largely unknown, but may include host genetic factors. This study aims to assess seropositivity, map antibody trajectories, and identify determinants of seropositivity and seropersistence.

**Methods:**

We analyzed anti-chlamydial immunoglobulin G levels in serum of women of reproductive age who participated in a prospective cohort and CT screening study. CT history was determined using screening results and self-reported diagnoses from sexual debut onward. We assessed seropersistence for n = 1,405 participants with samples at baseline and after six years, and seropositivity for n = 2,997 participants with baseline samples. Multivariable logistic regression identified demographic, behavioral, and host single nucleotide polymorphism (SNP) factors associated with seropersistence and seropositivity.

**Results:**

Among seropositive women at baseline, 42.0% (n = 118/281) were seropositive at follow-up. Seropersistence was more often found in women who reported treated asymptomatic and symptomatic CT infections as compared to those who did not (aOR: 3.74, 95%CI: 1.75–8.15 and 4.79, 95%CI: 2.42–9.47, respectively). Other associated factors were higher baseline antibody titers, carrying SNPs in TLR2 (aOR: 3.06, 95%CI: 1.31–7.36) and TLR9 (2.09, 95%CI: 1.09–4.08) genes and practical education (aOR: 3.16, 95%CI: 1.56–6.64). Seropositivity (24.9%, n = 748/2,997) was associated with a CCR5 deletion (aOR: 0.65, 95%CI: 0.42–0.99).

**Conclusions:**

CT seropersistence was more often found in women who reported treated CT infections as compared to women who did not report having had a CT infection or receiving treatment for it. Genetic predisposition and behavioral factors are linked to diversity in seropersistence patterns.

## Introduction

*Chlamydia trachomatis* (CT) infection is the most common bacterial sexually transmitted infection (STI), with an estimated 129 million new infections worldwide in 2020 [1]. It can be transmitted through vaginal, oral, and anal sex. In women, CT infection can lead to reproductive complications such as pelvic inflammatory disease (PID) and infertility in women [2]. In the Netherlands, prevalence in the general female population is estimated to range between 3% and 10% [3]. CT surveillance and monitoring is vital, yet it presents significant challenges due to the high prevalence of asymptomatic cases, which account for up to 75% of infections in women and often remain undiagnosed and unreported [4]. Diagnosis relies on nucleic acid amplification tests (NAATs), which only detect presence of CT DNA and necessitate testing within a specific timeframe.

Currently, in some settings it is proposed to limit asymptomatic CT screening because of limited public health benefits [5,6]. Additionally, in low-resource settings, NAAT availability is limited, hindering accurate disease burden estimates. Serology presents a viable solution, as it addresses the issue of missed infections associated with NAAT [7]. Unlike NAATs, serological assays allow for the estimation of lifetime exposure and detection of infections at various anatomical sites, including extragenital and upper genital tract locations that are not routinely sampled in NAAT diagnostics [8]. Additionally, blood samples can be conveniently sourced from existing biobanks not originally intended for STI research, facilitating broader surveillance efforts [9,10].

However, very few longitudinal studies have been done to assess seropersistence [11], which are essential for accurately interpreting estimates from serosurveillance. The relationship between CT infection history and serostatus remains unclear since not all people seroconvert, and antibody levels can decrease over time [12,13]. Factors influencing seroconversion, such as increasing number of sexual partners, being female, and older age, are largely understood [14–18], but there is a lack of studies examining factors associated with seropersistence. Specifically, research is needed to explore the associations between different methods of CT recovery, such as natural clearance versus antibiotic treatment, and their impact on seropersistence. Seropersistence may reflect the presence of functional antibody responses, including neutralizing antibodies that contribute to protective immunity [19].

Cellular immune responses triggered by infected host cells play a crucial role in clearance and recovery of CT infection [20]. Persons with an adequate innate immune response may clear CT infections rapidly, limiting adaptive immune response and resulting in minimal serological reaction. Twin studies suggest that up to 40% of the cellular immune response to Chlamydiae could be linked to host genetic factors [21]. Single nucleotide polymorphisms (SNPs) in pathogen recognition receptor (PRR) candidate genes, previously associated with CT pathogenesis [22], might therefore correlate with seropersistence.

This study has three aims: 1) to assess CT immunoglobulin G (IgG) seropositivity among women from a Dutch population cohort, both with and without a previous CT diagnosis, 2) to map changes in antibody trajectories over time; and 3) to identify demographic, behavioral and host genetic factors associated with CT seropersistence and seropositivity.

## Methods

### Study design and population

Data was used from a longitudinal cohort study (NECCST) following persons assigned female at birth (hereafter referred to as “women”) of reproductive age in the Netherlands prospectively for up to 14 years [23,24]. NECCST is a follow-up study from the Chlamydia Screening Implementation (CSI) study, which was performed between 2008 and 2011. For the CSI study, a population-based sample of sexually active participants (aged 16–29 years) was recruited from the general population in two major cities (Amsterdam and Rotterdam) and one semi-rural region (South Limburg) [25]. During the CSI participants with CT-positive results were referred to general practitioners or sexual health centers to receive treatment with antibiotics. Women from the CSI study, who agreed to participate in follow-up studies, were recruited between October 28^th^ 2015 and July 10^th^ 2016. Participants provided written consent to participate in the study and for their data to be published.

**Fig 1:**
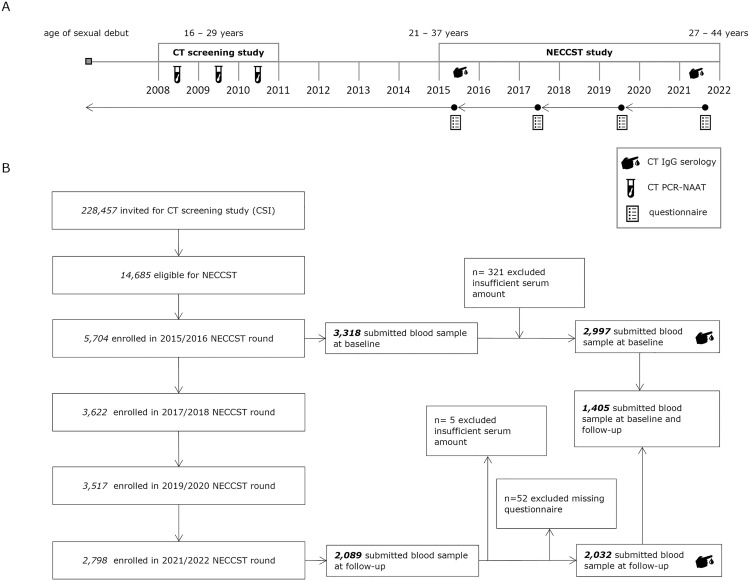
Study design and inclusion. (A) During the baseline questionnaire, participants were asked to provide retrospective information starting from their sexual debut. Consecutive questionnaires asked backed until the last previously filled in questionnaire. (B) Study inclusion flowchart. CT = *Chlamydia trachomatis*. CSI = Chlamydia Screening Implementation. NECCST = Netherlands Chlamydia Cohort Study.

NECCST consisted of four bi-annual data collection rounds. Questionnaires were sent out in 2015/16, 2017/18, 2019/20 and 2021/22. Participants who participated in the first round were re-invited via e-mail for each round unless they withdrew from the study. In the first and last round self-collected blood samples were collected to determine CT antibodies. For each participant at least one prior CT NAAT-PCR result was available from the CSI study, referred to as screening-PCR (Fig 1).

### Data

#### Questionnaires.

The online questionnaires contained questions on demographics, health, STIs, sexual behavior, pregnancy, fertility and long-term complications (including pelvic inflammatory disease (PID), tubal factor infertility (TFI) and ectopic pregnancy (EP). During the baseline questionnaire, participants were asked to provide retrospective information starting from sexual debut about experienced CT infections, pregnancies, and long-term complications. If the participant reported a previous or current CT infection, the year of diagnosis was documented, as well as symptoms related to the infection (altered vaginal discharge, vaginal bleeding, dysuria, pain during sex, lower abdominal pain) and whether they were treated with antibiotics.

#### Laboratory methods.

Participants received at baseline and at follow-up, to self-collect a capillary blood sample via finger-prick, which they returned via postal mail to the laboratory (a validated process [23,26]). Blood was collected in a serum separator tube (Becton, Dickinson and Company, USA). Upon receipt, samples were immediately centrifuged and serum was stored at −20°C until measurement in 2022. Chlamydia IgG antibodies were determined using an enzyme-linked immunosorbent assay based on conserved, serovar-independent and species-specific, recombinant Major Outer Membrane Protein (MOMP) antigen domains of CT (Serion Immunologics, Würzburg, Germany). The assay has demonstrated a sensitivity of 95.1% and specificity of 98.4%, as compared to another widely used MOMP-based CT IgG assay (CT IgG ELISA plus, Medac GmbH) [27].

Serological outcomes were classified based on concentration as positive (>15 AU/ml), negative (<10 AU/ml) or grey-zone (10–15 AU/ml) according to manufacturer’s instructions. Grey-zone outcomes were retested (grey-positive assigned positive; grey-grey assigned negative; grey-negative assigned negative). When the amount of serum was insufficient for a retest, samples were excluded (n = 326) (Fig 1).

DNA for SNP determination was extracted from buccal swabs, vaginal swabs, or urine. SNPs were analyzed using Kompetitive Allele Specific PCR [28].

### Definitions

#### Outcomes.

Seropositivity was analyzed cross-sectionally, at baseline (n = 2,997). Seropositive was defined as having a positive result from the serology assay, and seronegative as having a negative result.

Seropersistence was analyzed using the longitudinal data over 6 years (n = 1,405). Participants who provided a repeated sample were divided into four groups according to their antibody trajectory between baseline and follow-up: 1) negative-negative, 2) negative-positive, 3) positive-negative, and 4) positive-positive. We labelled “positive-positive” as seropersistence and “positive-negative” as waning.

#### CT status.

CT history was defined based on prior diagnosed CT infections: positive screening-PCR results or self-reported diagnoses in the questionnaire indicated as “CT+ history”, while negative screening-PCR results and no self-reported infections indicated as “CT- history”. Serostatus was **not** included in CT history. For women with CT+ history we assessed years since last CT infection (0–5 years/ 6 + years) and number of CT infections (1 infection/ 2 + infections).

Other factors related to CT were combined into categories based on reported infections, treatment and symptoms in the questionnaires. We took into account information from sexual debut until either baseline (cross-sectional data) or follow-up (longitudinal data). For symptoms and treatment we defined the following categories: 1) no CT or untreated asymptomatic (no reported CT or reported CT with no antibiotic treatment and no symptoms) 2) treated asymptomatic (reported CT treated with antibiotics, did not report any symptoms) and 3) treated symptomatic (reported CT treated with antibiotics, reported CT with symptoms) (Table 1).

**Table 1:** Classification of an individual’s CT status based on previous infections from screening and/or questionnaire responses. Serostatus is not included in the CT status.

Term	Definition
**CT history**	1) CT+ history: Positive screening-PCR results or self-reported CT diagnoses.
	2) CT- history: Negative screening-PCR results and no self-reported infections.
**Years since last CT infection** ^**#**^	1) No reported CT and no positive screening-PCR results
	2) 0–5 years: Last CT infection occurred within the past 5 years.
	3) 6 + years: Last CT infection occurred more than 6 years ago.
**Number of CT infections** ^**#**^	1) No reported CT and no positive screening-PCR results
	2) 1 infection: Individual has reported only one CT infection.
	3) 2 + infections: Individual has reported two or more CT infections.
**Ct infection symptoms and treatment** ^**#**^	1) No CT or untreated asymptomatic: No reported CT or reported CT with no antibiotic treatment and no symptoms.
	2) Treated Asymptomatic: Reported CT treated with antibiotics, without any reported symptoms.
	3) Treated Symptomatic: Reported CT treated with antibiotics, with reported symptoms.

^#^This variable was not included in the stratified models for seropositivity for the group with a CT- history. Abbreviations: CT = *Chlamydia trachomatis*.

#### Other factors.

The following potential factors were considered at baseline: age, migration background classified as ‘Western’ if both parents had a Western country of birth (i.e., country from Europe (excluding Turkey), North-America, Oceania, Indonesia, and Japan), ‘non-Western’ if at least one parent had a non-Western country of birth, and ‘unknown’ if one parent was Western whereas the country of birth of the other parent was unknown, or when country of birth of both parents was unknown. Educational level was categorized by theoretical and practical (theoretical—higher professional education and university education; practical—all other education levels).

The following lifestyle factors were included at baseline for cross-sectional data and follow-up for longitudinal data: Body mass index (BMI) (underweight <18.5/ healthy 18.5–25/ overweight 25–30/ obese 30 + kg/m^2^), and smoking (never/ former or occasional/ daily). Characteristics on sexual risk behavior were: age at sexual debut (≤15 years/ 16–17 years/ 18 + years), number of lifetime sex partners (<8/ 8–15/ 15+), number of lifetime CT tests (1-2/ 3-4/ 5+).

SNPs were assessed for PRR candidate genes previously associated with the immune response to CT [22], namely TLR2 (*rs4696480* and *rs5743708*), TLR4 (*rs4986790*), TLR5 (*rs5744168*), TLR9 (*rs187084*), NOD1 (*rs6958571*) and CCR5delta32 (*rs333∆deletion*). We included three types for each SNP (reference, single mutation, double mutation). K-nearest-neighbors was used to impute one or more missing SNPs (for 14.3% of participants, S1 Table).

### Statistical analysis

Study participant characteristics were described using descriptive analyses. Pearson’s Chi-squared and Fisher’s exact tests compared participants with follow-up samples across trajectories. The Kruskal-Wallis test assessed differences in baseline antibody titers between those with persistent versus waning antibodies.

Univariable and multivariable logistic regression were performed for factors associated with seropersistence and seropositivity. All candidate variables were initially included in the full model, and backward stepwise selection was applied using the Akaike information criterion (AIC). This approach iteratively removes variables if doing so improves the overall model fit by lowering the AIC. Migration background and age were included a priori in the multivariable models because of their established association with seropositivity [16]. Results were considered statistically significant when *p-*value ≤0.05. Odds Ratios (ORs) and adjusted Odds Ratios (aORs) with 95% confidence intervals (CIs) were presented. Prior to regression analysis, correlation of the variables was assessed to prevent multicollinearity. Before regression analysis, multicollinearity was assessed using generalized variance inflation factor (GVIF) scores, with a threshold of 1.5 for merging or exclusion. Since all scores were below 1.5, no variables required modification.

We analyzed two models:

1) To study factors of seropersistence, participants with persistent antibodies were compared to participants with waning antibodies. BMI was collapsed into two categories (underweight – healthy (<25)/ overweight – obese (≥25)). From the factors related to CT, only symptoms and treatment of CT infection was included in the multivariable model due to correlation with years since last CT infection and number of CT infections (these two factors were excluded).
2) We compared seropositive participants to seronegative participants at baseline. This analysis was stratified by CT history to distinguish between factors that could be associated with not seeking testing for CT infection (serology+ and CT history-) and those which are only associated with developing antibodies after a diagnosed CT infection (serology+ and CT history+).

We assessed if factors associated with seropersistence (model 1) are also associated with seropositivity (model 2). Statistical analyses were performed using R Statistical Software (version 4.2.0; R Foundation for Statistical Computing, Vienna, Austria) and the ‘VIM’ and ‘stats’ packages.

### Ethics statement

Participants provided written consent to participate in the study and for their data to be published. The study design was approved by the Medical Ethical Committee Noord-Holland, Alkmaar the Netherlands. In 2018 this committee ceased to exist, after which the approval of this study was taken over by the Medical Ethical Committee VU medical Center, Amsterdam the Netherland (NL 51553.094.14/2015.903(A2019.336)). Date of approval 10/13/2015. Dutch Trial Register NTR-5597.

## Results

### Study population

We included 2,997 women at baseline and 1,405 women at follow-up. Data from women with incomplete questionnaires at follow-up were excluded (n = 52) (Fig 1).

### Characteristics

Characteristics of women with repeated samples (n = 1,405) stratified by antibody trajectory are presented in Table 2. Median age at follow-up was 33 years (IQR: 30–36 years). The majority was of Western background (87%) and had a theoretical education (86%). The majority of women (55%−70%) had ≤ 1 new sex partner(s) in the last 6 years. Only 2.9% (n = 41) of women reported having a first CT diagnosis between 2015 and 2022. The percentage of women with a long-term sequelae was similar between the positive-positive and positive-negative group. Women with samples in both rounds showed comparable CT+ history (24% vs. 26%), education levels, and lifetime sex partners, to those with only a baseline sample (Table 2, S2 Table).

**Table 2:** Participant characteristics stratified by CT antibody trajectory. Characteristics (n (%)) of participants for longitudinal analysis (n = 1,405) stratified by CT antibody trajectory. Lifestyle characteristics and factors related to CT are assessed at follow-up unless otherwise specified.

Antibody trajectory between baseline (t = 0 years) and follow-up (t = 6 years)						
	Overall	Negative – negative	Negative – positive	Positive – negative	Positive – positive	p-value
	N = 1,405	N = 1,065	N = 59	N = 163	N = 118	
*Demographics*						
**Age** ^¹^ median (IQR)	33 (30, 36)	33 (30, 36)	32 (28, 34)	33 (30, 36)	34 (29, 37)	0.02
**Age** ^¹^						0.1
T1: 27–34 years	412 (29%)	312 (29%)	23 (39%)	43 (26%)	34 (29%)	
T2: 35–39 years	524 (37%)	405 (38%)	22 (37%)	64 (39%)	33 (28%)	
T3: 39–44 years	469 (33%)	348 (33%)	14 (24%)	56 (34%)	51 (43%)	
**Migration background** ^²^						<0.001
Non-Western	145 (10%)	91 (8.5%)	8 (14%)	22 (13%)	24 (20%)	
Western	1,228 (87%)	954 (90%)	49 (83%)	136 (83%)	89 (75%)	
Unknown	32 (2.3%)	20 (1.9%)	2 (3.4%)	5 (3.1%)	5 (4.2%)	
**Education** ^³^						<0.001
Theoretical	1,212 (86%)	942 (89%)	46 (78%)	143 (88%)	81 (69%)	
Practical	192 (14%)	122 (11%)	13 (22%)	20 (12%)	37 (31%)	
*Lifestyle characteristics*						
**BMI**						0.2
< 25 Underweight – Healthy	1,108 (79%)	851 (80%)	47 (80%)	126 (77%)	84 (71%)	
≥ 25 Overweight – Obese	297 (21%)	214 (20%)	12 (20%)	37 (23%)	34 (29%)	
**Smoking**						<0.001
Never	576 (41%)	458 (43%)	18 (31%)	64 (39%)	36 (31%)	
Former/ occasional	730 (52%)	545 (51%)	36 (61%)	88 (54%)	61 (52%)	
Regular/ Daily	99 (7.0%)	62 (5.8%)	5 (8.5%)	11 (6.7%)	21 (18%)	
**Age at sexual debut**						0.3
≤ 15 years	353 (25%)	262 (25%)	20 (34%)	40 (25%)	31 (26%)	
16–17 years	549 (39%)	415 (39%)	26 (44%)	59 (36%)	49 (42%)	
18 + years	503 (36%)	388 (36%)	13 (22%)	64 (39%)	38 (32%)	
**Number of lifetime sex partners** ^⁴^						<0.001
T1: < 8	451 (32%)	368 (35%)	16 (27%)	49 (30%)	18 (15%)	
T2: 8–15	439 (31%)	333 (31%)	20 (34%)	48 (30%)	38 (32%)	
T3: 15 +	509 (36%)	359 (34%)	23 (39%)	65 (40%)	62 (53%)	
**Number of lifetime CT tests** ^⁵^						<0.001
1–2 tests	467 (33%)	379 (36%)	12 (20%)	50 (31%)	26 (22%)	
3–4 tests	533 (38%)	412 (39%)	20 (34%)	66 (40%)	35 (30%)	
5 + tests	404 (29%)	273 (26%)	27 (46%)	47 (29%)	57 (48%)	
**Prior CT infections**						<0.001
Prior CT infection known	336 (24%)	171 (16%)	27 (46%)	54 (33%)	84 (71%)	
No known CT infection, serology only	175 (12%)	0 (0%)	32 (54%)	109 (67%)	34 (29%)	
No prior CT infection	894 (64%)	894 (84%)	0 (0%)	0 (0%)	0 (0%)	
**CT infection symptoms & treatment** ^⁶^						<0.001
No CT or untreated asymptomatic	1,094 (78%)	907 (85%)	35 (59%)	113 (69%)	39 (33%)	
Asymptomatic treated	144 (10%)	85 (8.0%)	9 (15%)	22 (13%)	28 (24%)	
Symptomatic treated	167 (12%)	73 (6.9%)	15 (25%)	28 (17%)	51 (43%)	
*Factors related to CT ^*	***N = 336***	***N = 171***	***N = 27***	***N = 54***	***N = 84***	
**Years since last CT infection** ^⁶^						0.002
0-5 years	57 (17%)	26 (15%)	12 (44%)	5 (9.3%)	14 (17%)	
6 + years	279 (83%)	145 (85%)	15 (56%)	49 (91%)	70 (83%)	
**Number of CT infections** ^⁶^						<0.001
1 infection	250 (74%)	144 (84%)	20 (74%)	37 (69%)	49 (58%)	
2 + infections	86 (26%)	27 (16%)	7 (26%)	17 (31%)	35 (42%)	
*Long-term complications*						
**PID** yes n(%)	51 (3.6%)	31 (2.9%)	0 (0%)	12 (7.4%)	8 (6.8%)	0.004
**TFI** yes n(%)	16 (1.1%)	8 (0.8%)	0 (0%)	4 (2.5%)	4 (3.4%)	0.023
**EP** yes n(%)	16 (1.1%)	8 (0.8%)	0 (0%)	5 (3.1%)	3 (2.5%)	0.024
*Recent sexual risk*						
**Self-reported CT diagnosis between 2012 and 2015 n (%)**						<0.001
No	1,321 (94%)	1,032 (97%)	53 (90%)	142 (87%)	94 (80%)	
Yes	84 (6.0%)	33 (3.1%)	6 (10%)	21 (13%)	24 (20%)	
**Self-reported CT diagnosis between 2016 and 2022 n (%)**						<0.001
No	1,337 (95%)	1,035 (97%)	47 (80%)	155 (95%)	100 (85%)	
Yes	68 (4.8%)	30 (2.8%)	12 (20%)	8 (4.9%)	18 (15%)	
**First self-reported CT diagnosis after 2015 n (%)**						0.001
No	1,364 (97%)	1,038 (97%)	51 (86%)	160 (98%)	115 (97%)	
Yes	41 (2.9%)	27 (2.5%)	8 (14%)	3 (1.8%)	3 (2.5%)	
**Number of new partners since 2015 n (%)**						0.002
0–1 partner	978 (70%)	762 (72%)	33 (56%)	111 (68%)	72 (61%)	
2–5 partners	246 (18%)	174 (16%)	15 (25%)	37 (23%)	20 (17%)	
6 + partners	181 (13%)	129 (12%)	11 (19%)	15 (9.2%)	26 (22%)	

P-value based on Wilcoxon rank sum test; Pearson’s Chi-squared test; Fisher’s exact test. ^ Chlamydia specific characteristics based only on participants with a known prior infection

Abbreviations: SNP = single nucelotide polymorphism, BMI = Body Mass Index, CT = Chlamydia trachomatis, PID = Pelvic inflammatory disease, TFI = Tubal factor infertility, EP = Ectopic pregnancy, NECCST = Netherlands Chlamydia Cohort Study, CSI = Chlamydia Screening Implementation study

¹ Determined at follow-up (t = 6 years)

² Migration background was classified as ‘Western’ if both parents had a Western country of birth (i.e., a country from Europe [excluding Turkey], North-America, Oceania, Indonesia, and Japan), ‘non-Western’ if at least one parent had a non-Western country of birth, and ‘unknown’ if one parent was Western whereas the country of birth of the other parent was unknown, or when country of birth of both parents was unknown.

³ Educational level was categorized by practical-educational level versus theoretical-educational level. Practical—no education, primary education only, lower general secondary education, and vocational education, Theoretical—higher professional education and university education. Missing 1.

⁴ Cumulative number of partners from sexual debut tot last follow-up. Missing 6.

⁵ Cumulative number from sexual debut tot last follow-up. Missing 1.

⁶ Based on screening-PCR and self-reported infections.

Characteristics of women at baseline (n = 2,997) stratified by seropositivity are presented in S2 Table. Overall seroprevalence was 25.0% (n = 748). More seropositive women reported first sexual contact ≤15 years compared to seronegative women (30% versus 25%). The majority of women had ≥ 8 lifetime sex partners (68%), with higher proportions among seropositives versus seronegatives (72% versus 66%). PID and EP were more often reported in seropositive women (5.5% versus 3.2% and 2.8% versus 0.8%, respectively). We identified 370 women with positive serology but CT- history, accounting for 32.3% (370/1,144) of all identified with CT (by either serology, screening-PCR or self-report).

### CT antibody trajectories

**Fig 2:**
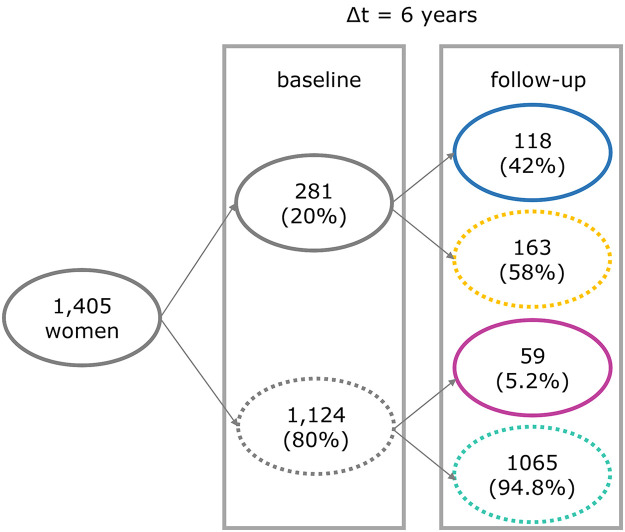
Overview of CT antibody trajectories from baseline to follow-up. Number (N) and percentage (%) are presented. Solid circle = seropositive, Dashed circle = seronegative. CT = Chlamydia trachomatis.

Among the 1,405 women, 42.0% of those initially seropositive were seropersistent, while 58.0% waned. For those seronegative at baseline, 94.8% remained negative, and 5.2% seroconverted at follow-up (Fig 2). Among women with a CT+ history, 25.0% were seropersistent compared to 3.2% in those with CT- history (Fig A in S1 File). Overall, the positive-positive group had significantly higher median antibody titers at baseline compared to the positive-negative group (p-value <0.001) (Figs B and C in S1 File). For both women CT+ history and CT- history, women with a positive-positive trajectory had a higher initial antibody titer compared to women with a positive-negative trajectory (p-value <0.001 for both strata of CT history) (Fig D in S1 File).

### Identified factors

**Fig 3:**
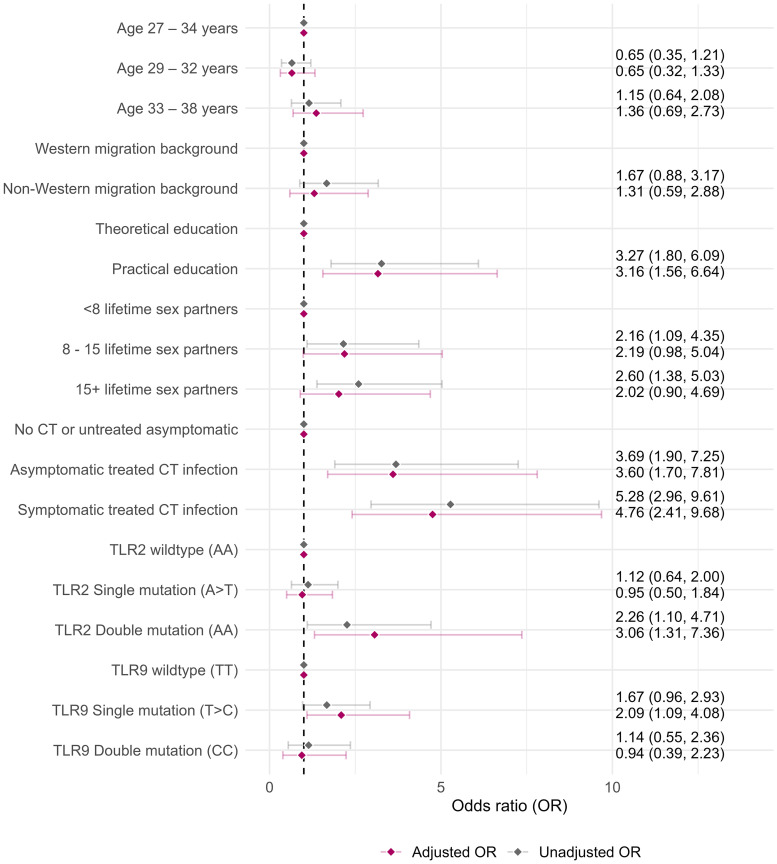
Factors included in multivariable logistic regression analyses for factors of seropersistence compared to non-persistent seropositivity. Complete analyses for alle factors can be found in S3 Table. CT = *Chlamydia trachomatis.*

Factors for seropersistence were practical education (aOR: 3.16, 95%CI: 1.56–6.64), asymptomatic treated CT infection(s) (aOR: 3.60, 95%CI: 1.70–7.81) and symptomatic treated CT infection(s) (aOR: 4.76, 95%CI: 2.41–9.68). Having 8–15 and 15 + lifetime sex partners were associated with seropersistence in the univariable model but this association did not remain significant in the multivariable model (aOR: 2.19, 95%CI: 0.98–5.04 and 2.01, 95%CI: 0.90–4.69, respectively). Furthermore, women carrying SNPs in the TLR2 gene and TLR9 gene were more likely to have persistent antibodies (aOR: 3.06, 95%CI: 1.31–7.36 and aOR: 2.09, 95%CI: 1.09–4.08) (Fig 3, S3 Table).

**Fig 4:**
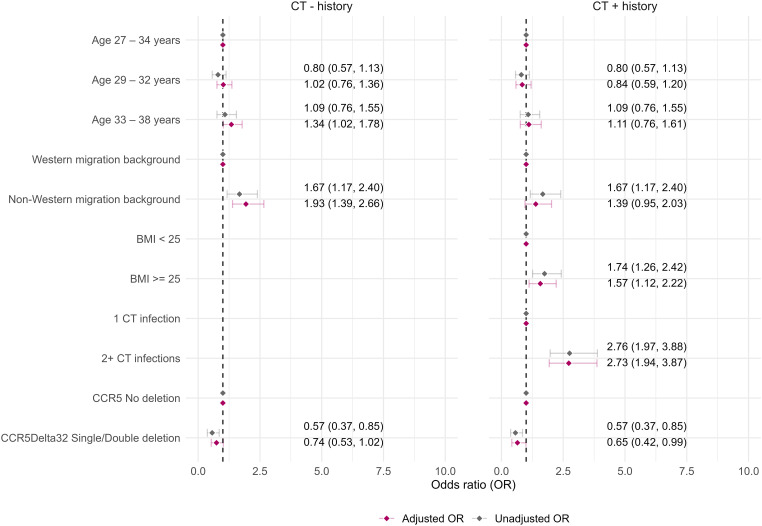
Factors included in multivariable logistic regression analyses for seropositivity stratified by CT history. Complete analyses for alle factors can be found in S4 Table. CT = *Chlamydia trachomatis.*

Factors for seropositivity were assessed separately for women with a CT+ and CT- history (Fig 4). For both history groups, unknown migration background was associated with seropositivity. Carrying a SNP in the CCR5 gene was associated with seropositivity in univariable analyses in both CT history groups, but only remained significant for women with a CT+ history in multivariable analysis (aOR: 0.65, 95%CI: 0.42–0.99). In women with a CT- history, Non-Western migration background was associated with seropositivity. Among those with a CT+ history, BMI ≥ 25 (overweight/obese) was associated with seropositivity. Moreover, having two or more diagnosed CT infections was associated with increased seropositivity (Fig 4, S4 Table).

None of the factors were associated with both seropersistence and seropositivity (Figs 3 and 4).

## Discussion

In this large population-based study among women of reproductive age, 42% of CT-seropositive women had persistent CT antibodies after six years. Seropersistence was four-to-five times more common among individuals who reported a CT infection treated with antibiotics compared to those who did not report having had a CT infection or receiving treatment for it. Among women who reported treated CT infections, seropersistence was lower in those with asymptomatic infections compared to those with symptomatic infections. The study also provides evidence linking behavioral factors and host genetic variations within candidate genes for pathogen recognition receptors to seropersistence (*TLR9* and *TLR2*) and seropositivity (*CCR5delta32*).

This study was based on a long-term prospective cohort of almost 3000 women with detailed data on covariates. Some limitations need to be considered. First, the ELISA assay used focuses specifically on MOMP, thereby potentially missing past CT infections that could have been identified by using for example mixed peptide assays [29]. The probability of negative serology is smallest in those with repeated exposure [11,13,30], which could lead to an underestimation of positivity and persistence in those with low risk behavior or limited exposure. Second, it was assumed that seropositivity in both sample rounds means persistence. Yet, because study subjects were only sampled twice at fixed time points, antibody responses to repeat infections may have been missed. However we expect this to be minimal based on biyearly self-reported CT infections and sexual behaviors in the period between sampling intervals, which show high baseline and low recent sexual risk behavior. Finally, the second sample was collected at in women with a median age of 33 years, thus results may have limited generalizability to persistence patterns directly after exposure or in current highly exposed populations such as young women.

Previous studies have shown a substantial decline in antibody levels in the first 6 months post-infection [30], followed by a stable phase that can persist for up to 12 years after infection [11,17]. Our rates are in accordance with a study in pregnant women that used a similar MOMP-specific assay and calculated 48.4% seropersistence 5–6 years after baseline seropositivity [11]. In comparison, studies on CT antibody persistence with ELISA assays based on the CT-specific Pgp3 protein established higher proportions of seropersistence compared to our study. For example, *Horner et al.* established that over 95% of seropositive women aged 26 years recruited at UK clinics were still seropositive after 12 years [17]. Assay differences likely stem from the chlamydial life cycle. The MOMP protein, surface-localized and shielded during replication, contrasts with the Pgp3 protein, which is secreted into the host cytosol, making it potentially more easily picked up by the host immune response and thus better detectable by serology assays [31]. However, the Pgp3 assay is not yet commercially available and thus public health application is limited due to the lack of certification.

Our study is the first to separately assess antibody patterns over time in treated asymptomatic and treated symptomatic individuals, and compare them to those who did not report having had a CT infection or received treatment for it. Likely, asymptomatic infections were milder and prompted minimal antibody response, which could be caused by a combination of pathogen and host factors [11]. Our results partly correspond to a recent modelling study by *Clay et al.*[32] suggesting symptomatic infections increase seropersistence. This is expected as symptoms are usually a sign of an activated immune response after bacterial infection. Furthermore high levels of antibodies are found more often in women with complications like PID and infertility due to enhanced inflammation and tissue damage [32–35].On the other hand, it has been hypothesized that natural clearance is more likely to lead to long-term seroconversion [32], due to treatment interrupting the development of a specific antibody response [36,37]. Treatment may interrupt the development of antibodies that play a role in resolving re-infection and, to a lesser extent, primary infection. These antibodies act as facilitators of both the adaptive and innate immune responses [38]. Our results showed that seropersistence was more common in women with treated infections compared to untreated infections. This is in line with prior studies that showed that high seropersistence of CT antibodies can be found even after treatment with antibiotics [11,13,17]. An explanation for this result might be residual confounding that resulted from unmeasured behavioral risks associated with both (repeated) exposure, severity and persistence. Another explanation might be a biological effect in which previous treatment results in a subsequent infection being less easy to clear and possibly more severe as the prior immune response has not been adequately [36,37]. This could then be reflected in increased seropersistence among those treated with antibiotics.

Practical education was associated with seropersistence and might reflect more risky sexual behaviors. Being overweight/obese (BMI ≥ 25) was associated with seropositivity among women with a CT+ history, potentially due to the effects of chronic inflammation and impaired immune regulation linked to obesity [39]. These factors can enhance antigen exposure and increase the likelihood of seroconversion in response to chlamydia infection. Alternatively, BMI could represent unmeasured confounders related to sexual behavior. The effect of sexual risk behavior on seropositivity and seropersistence could be due to increased exposure or a higher risk of complicated infections, both of which might boost the immune response by elevating antibody levels [40]. Seropositivity in women with and without a CT+ history was associated with an unknown or non-Western migration background, which besides a proxy for behavior, socio-economic status or sexual networks could be related to differential immune response to CT across ethnic groups [16,41]. Women with two or more CT reported infections were more likely to be seropositive, which is in agreement with previous research [11,13,30]. Only among women without a previous CT diagnosis (CT- history), older age was a factor for seropositivity. It could be that these women had a higher CT exposure or were more likely to have an undiagnosed infection due to limited CT testing and treatment when they reached the age of sexual activity [18].

Several SNPs were associated with seropersistence or seropositivity. The SNPs studied were previously linked to CT infections by either clinical studies and/or murine studies focusing on their potential impact on gene and protein expression or function [22]. It is hypothesized that women with an adequate innate immune response clear CT infections at a rate that limits the development of an adaptive immune response, leading to minimal serological reaction. Our findings align with previous studies that find that toll-like receptors are involved with susceptibility to and the severity of infection and an effective host defense against Chlamydiae [42–45]. Seropersistence was associated with carrying common SNPs in *TLR2* (*rs4696480* AA) and *TLR9* (*rs187084* T > C) genes. This could be explained by the development of symptoms and predisposition to complications such as tubal pathology [42,43,45]. Furthermore there are studies linking *TLR9* (*rs187084* T > C) to cervical cancer risk possibly explained by HPV infections changing the expression of toll-like receptors [46]. In addition, we find that women with the CCR5delta32 allele (allelic frequency around 10% in Caucasians) are less likely to develop antibodies after CT infection. This allele codes for a truncated and non-functional chemokine receptor CCR5 protein variant [47]. Individuals with one copy of CCR5delta32 are somewhat resistant to HIV, while those with two copies are almost completely immune [48]. For CT, this is the first observational study in line with the hypothesis from mouse-models that CCR5 deletions may result in delayed resolution of genital infections [49]. Larger studies in populations in which CCR5 deletions are more frequent are needed to confirm this observation [50].

Ultimately, relying on CT serology to estimate population-wide risk may overlook women with lower sexual risk behavior and untreated CT infections. Understanding how to interpret serology is crucial, especially when designing surveillance or prevention strategies based on such estimates. If serological assays are considered in future vaccine development to measure antibody responses as a proxy for vaccine-induced immunity, it is vital to take into account potential host genetic predispositions. Our data furthermore provide an argument for research into to effects of treatment for asymptomatic infections, as diversity of seropersistence patterns between individuals could be a proxy for varying treatment response, pharmacokinetics and ultimately treatment benefits.

## Supporting information

S1 Table Missing data for single nucleotide polymorphisms (SNPs). Numbers and proportions (n %) of participants with missing SNPs and resulting SNP distributions after imputation.
(XLSX)

S2 Table Participant characteristics stratified by seropositivity. Characteristics of participants for cross-sectional analysis (n = 2,997) at baseline separate for seronegative and seropositive participants.
(XLSX)

S3 Table Complete analyses of factors for seropersistence. Univariable and multivariable logistic regression analyses for factors of persistent CT IgG seropositivity compared to non-persistent seropositivity.
(XLSX)

S4 Table Complete analyses of factors for seropositivity. Univariable and multivariable logistic regression analyses for factors of persistent CT IgG seropositivity compared to non-persistent seropositivity.
(XLSX)

S1 File Antibody titers. (PDF)
