# Supplementary material for: Prevalence and risk factors for Chlamydia trachomatis seropositivity and seropersistence among women: A prospective cohort study
Source: PLoS One. 2025 Aug 5;20(8):e0328449. doi: 10.1371/journal.pone.0328449 (PMC12324120; doi:10.1371/journal.pone.0328449)

S1 File. Antibody titers

Figure A. Classification of women by CT antibody titers at baseline (t = 0 years) and follow-up (t= 6 years) for different CT history groups (n represent numbers per history group). Proportion of women in each of the antibody trajectories is indicated by colored percentages in the plots. CT history is defined by positive screening-PCR results or self-reported diagnoses in the questionnaire. History is stratified as (**A)** overall (**B**) negative CT history (**C**) positive CT History. Medians can be found in Figure S2. CT = *Chlamydia trachomatis*

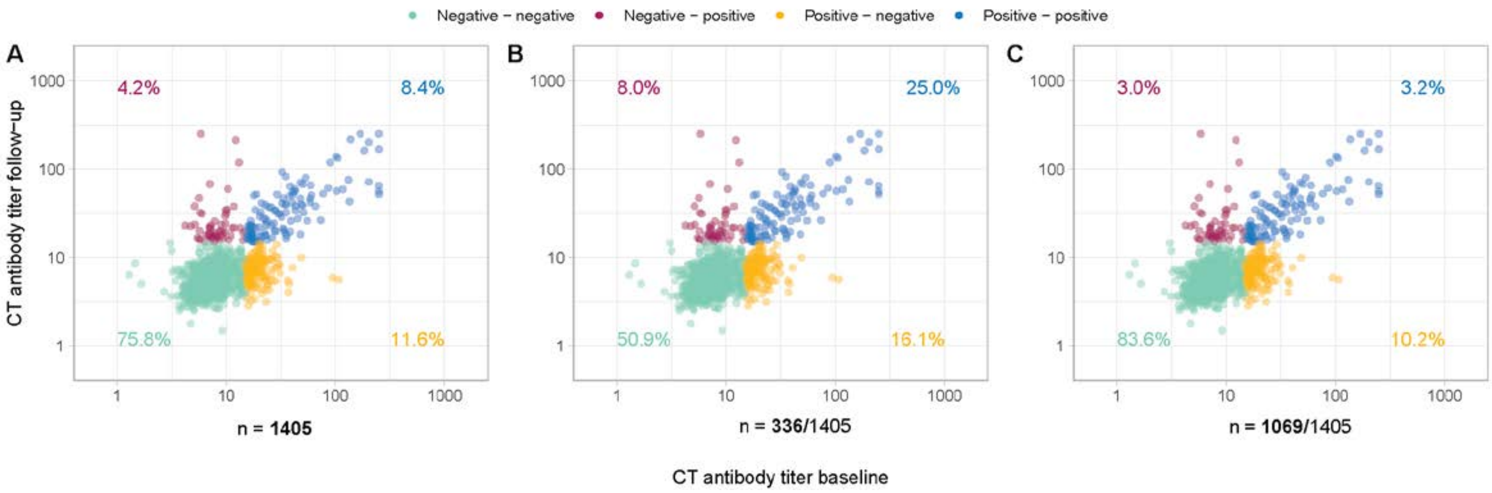

Figure B. CT antibody titer trajectories and distributions for women at baseline (t = 0 years) and follow-up (t= 6 years). Dotted horizontal lines indicate medians by at baseline and follow-up grouped by antibody trajectory. Statistical differences between medians can be found in Fig S3. CT = *Chlamydia Trachomatis.*

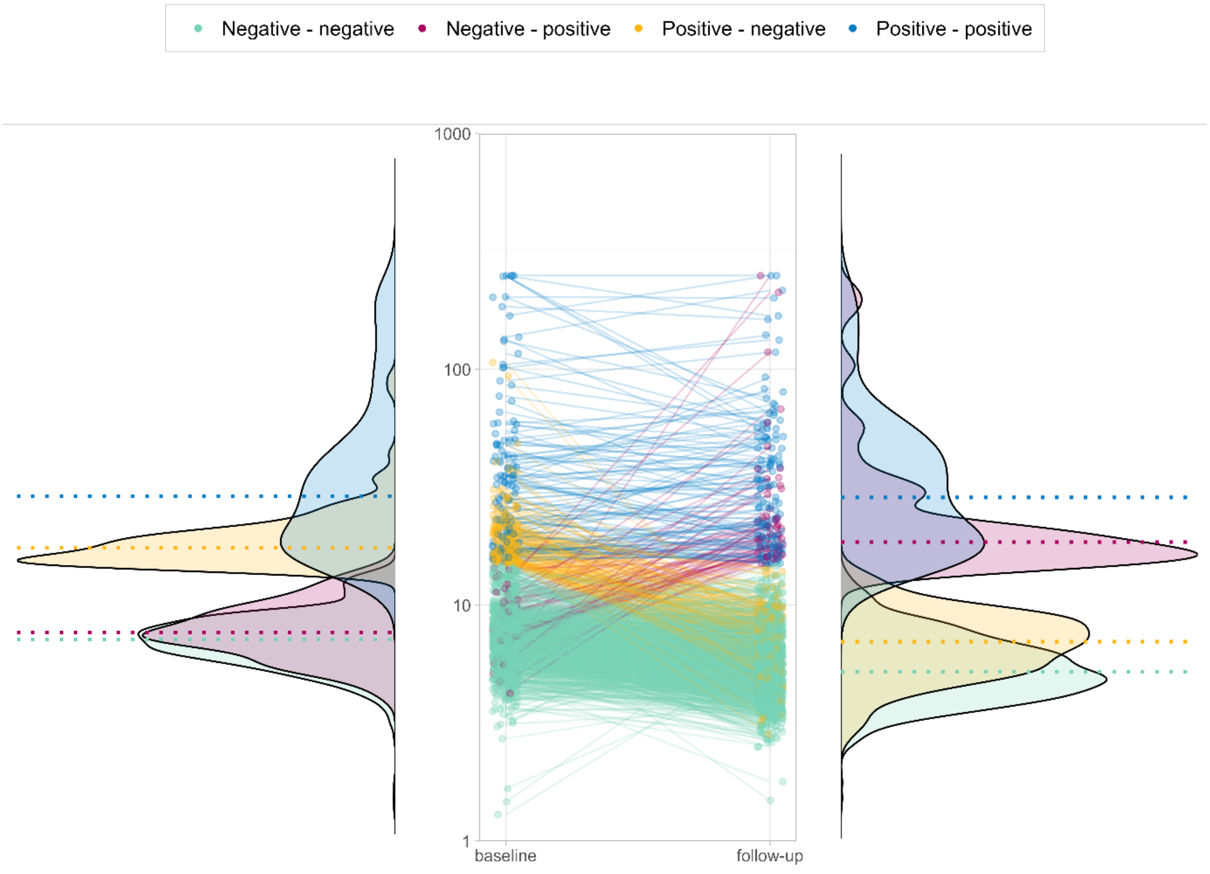

Boxplots of CT antibody titer levels

The line within the box corresponds to the median value of the group, the lower and upper lines of the box correspond to the first and third quartiles (25th and 75th percentiles). The whiskers extend to the smallest and lowest value 1.5 * IQR. The dots above and below the whiskers correspond to outliers. Grey boxes indicate the grey-zone of the serological assay as defined by the manufacturer (OD between 10 AU/ml - 15 AU/ml).

OD = optical density, IgG = Immunoglobulin G, AU = arbitrary unit, ml = milliliter, CT = *Chlamydia Trachomatis*

Figure C. Boxplot of longitudinal CT antibody titer levels (in AU/ml) per antibody trajectory.

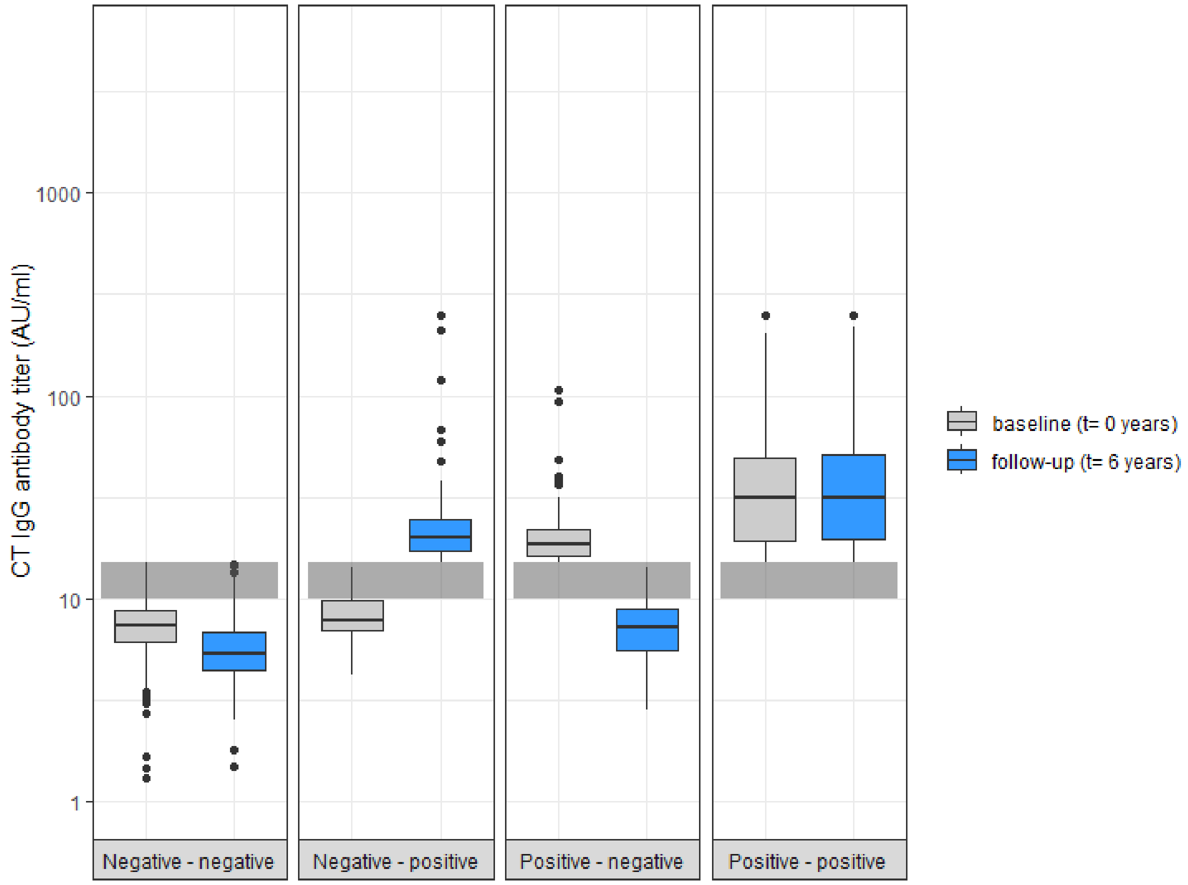

CT = *Chlamydia Trachomatis*

Figure D. Boxplot of baseline antibody titer levels for positive-negative (grey) and positive-positive (purple) trajectory groups. A) Overall B) Stratified by CT history: negative CT history (no diagnosed CT infection(s)) (left) and positive history (diagnosed CT infection(s)) (right). P-values represent Kruskal-Wallis rank sum test used for comparison.

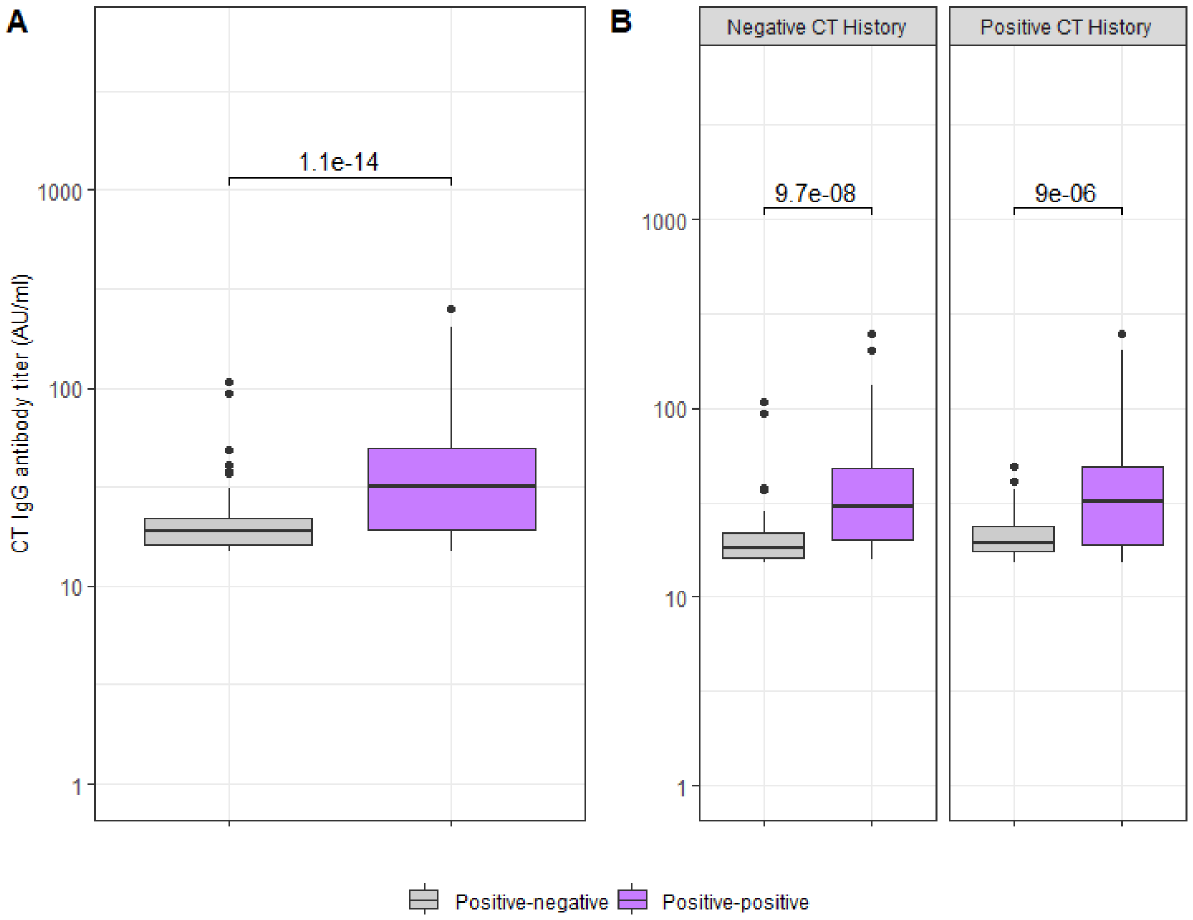

Supplement: S1 File — (PDF) [file pone.0328449.s005.pdf]
